# Decreasing Social Isolation to Enhance Mental Health among Older Adults in China: A Mediation Analysis of Aging Attitude

**DOI:** 10.3389/fpsyg.2021.735740

**Published:** 2021-09-24

**Authors:** Xinfeng Cheng, Theodore D. Cosco, Tolulope Ariyo

**Affiliations:** ^1^School of Economics and Management, Xi’an Technological University, Xi’an, China; ^2^Department of Gerontology, Gerontology Research Center, Simon Fraser University, Vancouver, BC, Canada; ^3^Institute for Population and Development Studies, School of Public Policy and Administration, Xi’an Jiaotong University, Xi’an, China

**Keywords:** social isolation, aging, mediation analysis, mental health, China

## Abstract

A large body of literature has examined the relationship between social isolation and mental health in older adults. However, only a few studies have examined the mediating effects of aging attitudes on this relationship. This study investigated the impact of objective isolation (family isolation, friend isolation, and community isolation), and subjective social isolation (perceived isolation) on the mental health of Chinese older adults, and the mediating effect of aging attitudes. Mental health was assessed through depressive symptoms, using the Epidemiological Studies Depression Scale. The research sample comprising 7,024 elderly adults (60 years old), was obtained from the nationally representative 2014 Chinese Longitudinal Aging Social Survey. The regression analysis indicated that objective social isolation and subjective social isolation are independently related to mental health among older adults. Furthermore, in the mediation analysis, aging attitude was found to play a significant mediating role between social isolation and mental health. Our study concludes that though, objective and subjective social isolation are issues affecting mental health in older people, however, aging attitude also needs to be factored in that relationship as we have shown that there is a significant mediating effect.

## Introduction

Five years ago in 2015, the population of China was 1.37 billion, and people aged 60 years or older constituted 16.6%. It is projected that by 2030, this will increase to 25% (United Nations, 2019). Old age may often be accompanied by failing health status (Robins et al., 2018), such that it may affect the physical, mental, or emotional state of mind of the elderly. Also, the reduced social participation that may come as an extended effect could exacerbate any physical, psychological, financial, or emotional difficulties (Kaye and Singer, 2018). According to estimations based on national household surveys, the prevalence of severe depression among Chinese older adults is 7.1%, while depression symptom is reported to be 37.9% (Qin et al., 2016). A higher prevalence of depression symptoms at 43.4% has also been reported among the group of older adults who are socially isolated (Huang et al., 2020).

Social relationships are important for mental and physical well-being all through one’s life span (Cacioppo and Cacioppo, 2014). The social convoy model suggests that social relationships can protect an individual’s health (Antonucci and Akiyama, 1995; Antonucci et al., 2014). Individuals with enhanced social relationships not only experience improved psychological well-being but also, better physical health (Cohen, 2004). The extent to which a person is socially isolated (objective social isolation), and the extent to which they feel socially isolated (subjective social isolation) are some of the ways of evaluating social relationships (Cacioppo and Cacioppo, 2014). Objective social isolation specifically relates to physical separation from other people or a deficiency in interaction (Taylor et al., 2018). On the other hand, subjective social isolation indicates one’s perception of the quality of relationships or interaction with others (Valtorta et al., 2016).

Social isolation has often been measured in an objective or subjective context. Individuals may experience loneliness without also suffering social isolation, or vice versa (Valtorta and Hanratty, 2012). The advancement of social isolation research over the years has helped scholars realize that both objective and subjective components of social isolation must be examined (Cho et al., 2019). It is important to differentiate between objective and subjective social isolation, particularly in how they are both associated with health. Knowing their specific contributions to health may shed some light on their relatedness or independence, and provide insight on possible interventions (Holt-Lunstad et al., 2015).

Social isolation affects mental health through psychological, behavioral, and physiological pathways (Holt-Lunstad et al., 2010). The psychological pathway has been established as an important linkage between social relationships and health (Berkman et al., 2000). For example, social isolation can cause low self-efficiency and self-respect (Umberson and Montez, 2010), reduced emotional support (Santini et al., 2017), and increase perceived lack of well-being (Berkman et al., 2000), which then affect the mental health of older people.

Aging attitude is a predictor of successful aging (Korkmaz Aslan et al., 2019). It refers to an individual’s cognition and opinion on the aging of oneself and others (Laidlaw et al., 2007), and the manner it affects the individual’s physical and mental health (Jang et al., 2004; Wurm et al., 2017). Previous studies have shown that a positive aging attitude among older adults can stimulate good self-rated health and better life satisfaction. Conversely, a negative aging attitude can lead to poor physical health, depression, and low subjective well-being (Diehl et al., 2014).

While it is generally perceived that an individual’s aging attitude is affected by factors such as age, gender, and socioeconomic status (Laidlaw et al., 2007; Diehl et al., 2014), social relationships, or more specifically, social support/isolation is also an important factor that may affect aging attitude (Tong and Lai, 2016; Santini et al., 2017). For instance, social support is likely to increase access to emotional (e.g., love and care) and instrumental supports (e.g., material assistance and information), both of which are germane to dissuading negative attitudes toward aging (Lamont et al., 2017). Furthermore, the model of Awareness of Aging (AoA) as proposed by Diehl et al. (2014), depicts aging attitude as an important mediator between social relationships and health and also emphasizing the influence of aging attitude on health. This goes to suggest that while aging attitude may have a direct link with mental health among older adults, it may also act as a potential mediator for any effect from social isolation.

Based on the avalanche of evidence in the literature, both objective and subjective aspects of social isolation may be similar or different in how they impact the mental health of older adults, and in light of the contradictory findings, researchers have often drawn contrary conclusions. For instance, some studies have found that older adults who experience objective social isolation are likely to be more depressed (Cornwell and Waite, 2009), and have a lower rating on life satisfaction (Thompson and Heller, 1990; Zebhauser et al., 2014; Courtin and Knapp, 2017). Conversely, other studies have reported that it is not objective social isolation, but rather, perceived isolation (that is, loneliness) that poses a risk for depression in older adults (Umberson et al., 2010; Lim and Kua, 2011; Taylor et al., 2018). Another study had yet, reported that both objective and subjective social isolation are significantly related to depression in older adults, emphasizing no difference in the sizes of effect (Ge et al., 2017). Additionally, while the conclusions from another study (Coyle and Dugan, 2012), is partly in agreement with Ge et al. (2017), their point of departure is that there is a difference in the sizes of effect. They posited that given the same magnitude of depression, older adults tend to have a higher degree of social isolation, and a lower degree of perceived isolation (Coyle and Dugan, 2012). Further evidence from Taylor et al. (2018), suggested that while the experience of objective isolation from relatives and friends is uncorrelated with depressive symptoms and psychological stress in older adults, the experience of subjective isolation from relatives and friends is correlated with depression and psychological stress.

From the perspective of the internal mechanism of the impact of social isolation on mental health in older adults, most existing studies have indicated that social isolation can reduce self-efficacy (Schrempft et al., 2019), and self-esteem (Umberson and Montez, 2010), decrease emotional support (Santini et al., 2015), and reduce perceived happiness (Berkman et al., 2000). Although various mechanisms have been verified to operate between social isolation and the mental health of older adults, few studies have considered that social isolation could affect the mental health of older adults through a psychological mechanism such as aging attitudes (Liu et al., 2020). While social isolation has been linked to the risk of morbidity and mortality (Laugesen et al., 2018), however, it has most commonly been associated with mental health-related issues, particularly among older adults (Gerino et al., 2017; Wang et al., 2017). According to the American Academy of Social Work and Social Welfare, social isolation is a major problem of special concern for older people (Kaye, 2017). Although there are lots of older people in China, only a few studies have investigated how social isolation impacts their mental health, concluding that social isolation is linked to mental health, and the links are independent of loneliness (Wu and Sheng, 2020; Yu et al., 2020).

Against the backdrop of Confucian ideology, the Chinese culture is heavily characterized by the principles of collectivism, emphasizing family togetherness and the interdependency of relationships. In such a context, families, friends, and acquaintances provide different types of social support to the Chinese elderly. By implication, isolation from family, friends, and acquaintances may have adverse effects on Chinese older adults. Therefore, the main objective of this study is to investigate how forms of social isolation are associated with the mental health of older adults, using depression symptoms as a measure. Also, to examine how the attitude to aging plays a mediating role in that relationship. In this study, objective social isolation has been divided into family isolation, friend isolation, and community isolation. The following questions are of concern: (1) Is there an independent effect of objective and subjective social isolation on the mental health of older Chinese adults? (2) Are there variations in how the different forms of social isolation impact the mental health of older adults in China? (3) Is aging attitude a mediator of the relationship between social isolation and the mental health of Chinese older adults?

## Materials and Methods

### Data Set

We analyzed secondary data obtained from the 2014 Chinese Longitudinal Aging Social Survey (CLASS). The survey was conducted by Renmin University with the help of the Chinese government. It aimed to gather data from Chinese elderly adults aged 60 and above to identify their issues and challenges. The questionnaire included questions about social, economic, and physical well-being, among other topics. The survey protocol was approved by the Ethics Committee of the Renmin University of China. Data collection lasted from March to December 2014, and it followed a multi-stage sampling technique covering a total of 462 villages/neighborhood communities across 28 provinces (autonomous regions/municipalities). Data were collected through a face-to-face interview by trained interviewers. Each participant was informed of the purpose of the survey. Participation was voluntary, and participants were assured of the confidentiality of any information collected. Informed verbal consent was obtained from the participants before the questions were administered, and no financial compensation was given for participation. The survey response rate was reported to be 97%. More details about the survey procedure can be found in the survey final report (National Survey Research Center, 2014).

The total sample obtained for the survey was 11,511. Among these, 4,487 observations were excluded based on missing responses on key variables of interest. For this group, the average age was 72.6 years, showing that they were much older people. About 46.1% of them were widowed, 48.3% rural residents, and 62.3% women. Among these excluded samples, about 65.5% (2,943) was because they did not respond to questions on aging attitude due to low cognitive ability. The final sample included in our analysis was 7,024. Our comparison of the deleted and analyzed samples showed some differences. Compared to the excluded observations, the included observations had a better cognitive ability, younger average age, a larger proportion of males, a higher proportion of rural residents, and a lower proportion of widowed.

### Dependent Variable

In the current study, the dependent variable is depression. During the survey, this was measured through the revised Center for Epidemiological Studies Depression Scale (CES-D) which contained 9 items (Silverstein et al., 2006). Each item has three possible responses: “no” = 1; “sometimes” = 2; “frequently” = 3. The total score ranges from 9 to 27, with a higher score indicating more depressive symptoms. Some examples of the items on the scale are: “Do you think you are in a good mood?,” “Do you feel lonely?,” and “Do you think you are useless?.” The Cronbach alpha reliability coefficient of the scale for the current study is 0.755.

### Key Independent Variables

The key independent variable is social isolation, which was divided into objective social isolation and subjective social isolation.

#### Objective Social Isolation

Objective social isolation was divided into “family isolation,” “friend isolation,” and “community isolation.”

##### Family Isolation

Family isolation was measured using the Lubben Social Network Scale (LSNS) (Lubben et al., 2006), which has been cross-validated for a Chinese context (Chang et al., 2018). The following items in the questionnaire relating to the family member network were used to measure the construct of family isolation. (1) “How many relatives do you see or hear from at least once a month?”; (2) “How many relatives do you feel at ease with that you can talk about private matters?”; and (3) “How many relatives do you feel close to such that you could call on them for help?” The participants had to choose from six options: “none” = 0; “one” = 1; “two” = 2; “three or four” = 3; “five to eight” = 4; or “nine or more” = 5. The responses to the three questions were aggregated to get the total score for family isolation. We used the cut-off score suggested by Lubben et al. (2006), which is “6 points,” meaning that older adults with a home network score of below 6 were considered to be in family isolation (1 = experienced family isolation), and those with a score of 6 and above were considered not to have experienced family isolation (0 = no family isolation). The Cronbach alpha reliability coefficient of the family isolation dimension in the current study is 0.797.

##### Friend Isolation

Friend isolation was also measured using the following items from the LSNS. (1) “How many of your friends do you see or hear from at least once a month?”; (2) “How many friends do you feel at ease with that you can talk about private matters?”; (3) “How many friends do you feel close to such that you could call on them for help?” The participants had to choose from five options: “none” = 0; “one” = 1; “two” = 2; “three or four” = 3; “five to eight” = 4; or “nine or more” = 5. Similar to family isolation, the responses to the three questions were aggregated to get the total score for friend isolation. Respondents with a friend network score below 6 were considered to be in a state of friend isolation (1 = experienced friend isolation), and those with a friend network score of 6 and above are regarded as not having experienced friend isolation (0 = no friend isolation). The Cronbach alpha reliability coefficient of the friend isolation dimension in the current study is 0.837.

##### Community Isolation

Based on Menec et al. (2019), community isolation was measured through one question item. “Did you participate in the following activities in the past three months?”: community security patrols, caring for other older people, environmental sanitation protection, dispute resolution, escort chat, voluntary services requiring professional skills, and help to look after other’s children. Each of the activities had the following response options: “Participated”; “I have participated, but not in the past three months”; “Never participated.” In this study, the total score was calculated by combining the responses to each activity. If the respondent chose “Never participated,” it was regarded as experiencing community isolation, while the other two options were regarded as not experiencing community isolation (0 = not experiencing community isolation; 1 = experiencing community isolation).

#### Subjective Social Isolation

In this study, subjective social isolation was conceptualized as “perceived isolation.” In the survey, questions bothering on this concept were based on the Hughes et al. (2004) revised three-item loneliness scale known for high-reliability of construct. The three questions are: (1) “In the past week, did you feel that you lacked companionship?”; (2) “In the past week, did you feel that you were ignored by others?” and (3) “In the past week, did you feel that you were isolated by others?” Each question had three response choices: “no” = 1, “sometimes” = 2, and “frequently” = 3. Aggregating the answers to the questions yielded the total score for loneliness, with a possible range from 3 to 9 points. Adopting the criterion of Shaw et al. (2017), a score of three or less was defined as no perceived isolation, and scores above 3 were considered to indicate perceived isolation (0 = no perceived isolation; 1 = perceived isolation). The Cronbach alpha reliability coefficient of the perceived isolation dimension in the current study is 0.821.

### Mediation Variable

The mediating variable in this study is “aging attitude.” In the survey, The Attitudes to Aging Questionnaire (AAQ) (Laidlaw et al., 2007), was used to elicit questions relating to the construct on aging attitude. The AAQ had been previously validated across multiple cultures and has often been used in studies on the Chinese context (Wang et al., 2009; Laidlaw, 2010). The AAQ uses a five-point response scale ranging from “completely disagree” to “completely agree” on the following seven items: (1) “I think I am old”; (2) “In my opinion, aging is a process of constant loss (such as loss of health, loss of friends and relatives, loss of ability, etc.)”; (3) “I found it harder to make new friends when aging”; (4) “For my age, I feel that I was excluded”; (5) “The older the person, the stronger the ability to deal with life problems”; (6) “Wisdom grows with age”; and (7) “There are many pleasant things about aging.” Reversing the order of the responses to the last three items and then adding the responses to all the items yielded the individuals’ “aging attitude.” The Cronbach alpha reliability coefficient of the aging attitude dimension in the current study is 0.752. The “aging attitude” variable was a continuous variable, with higher scores indicating a more positive aging attitude.

### Control Variables

Based on the factors that may affect the mental health of older adults, as have been reported in the literature (Thompson and Heller, 1990; Courtin and Knapp, 2017; Taylor et al., 2018; Huang et al., 2020; Liu et al., 2020; Wu and Sheng, 2020), the following variables were selected as control variables for this study: social and demographic characteristics, such as age, gender, marital status, education level, household registration attributes, working status, and living arrangements (“living alone” = 0; “live only with spouse” = 1; “live only with children” = 2; “live only with others” = 3). Additionally, the instrumental activity of daily living (IADL) (Lawton and Brody, 1988), indicating how well-respondents can cater to self needs was also part of the control variables. The IADL items in the 2014 CLASS covered sundry questions relating to shopping, cooking, telephoning, medicine, funds management, traveling, and doing chores. Furthermore, cognitive ability was part of the control variable and in the 2014 CLASS, it was measured through the mini-mental state examination (MMSE) scale (Folstein et al., 1975). Both the IADL and the MMSE had previously been validated and translated for a Chinese context (Chiu et al., 1994; Tong and Man, 2002). The selected control variables also include the number of children and religious faith (0 = No, 1 = Yes). The coding and operationalization of all variables in this study are depicted in Table 1.

**Table 1:** Coding and operationalization of variables.

**Variables**	**Definitions**
**Independent Variables**	
Objective social isolation	
Family isolation	1 = experienced family isolation;0 = no family isolation
Friend isolation	1 = experienced friend isolation;0 = no friend isolation
Community isolation	1 = experienced community isolation;0 = no community isolation
Subjective social isolation	
Perceived isolation	1 = perceived isolated;0 = no perceived isolated
**Dependent Variable**	
Depression	9–27
**Mediation Variable**	
Aging attitude	7–35
**Control Variables**	
Age (years)	60–113
Gender	0 = Female; 1 = Male
Education	0 = Elementary school and below; 1 = Junior high school; 2 = High school and above
Urban	0 = Rural area; 1 = Urban area
Married	0 = Married; 1 = No spouse (Widowed, Divorced, Unmarried)
Working	0 = No; 1 = Yes
Log of income (Chinese Yuan)	2.30–13.77
Religious faith	0 = No; 1 = Yes
Instrumental Activity of Daily Living (IADL)	7–20 **(**higher score represents better IADL**)**
Cognitive ability	3–16 (higher score represents better cognitive ability)
Living arrangement	0 = Live alone; 1 = Live only with spouse; 2 = Live only with children; 3 = Live with other people
Number of children	0–9

### Analysis

We used a multiple linear regression model to analyze the relationship between social isolation and mental health. Following this, structural equation modeling was used for mediation analysis, and the Bootstrap method was used to verify the mediation effects of aging attitudes.

To solve the sample selection bias and address the causal relationship of social isolation and mental health, the propensity score matching (PSM) method was used. The PSM is a technique appropriate for the estimation of the causal effect in a situation where treatment is non-randomized (Rosenbaum and Rubin, 1983). One major advantage of the PSM in such a context is that it can simulate a randomized trial, assigning individuals into treatment and control groups (Lian et al., 2011). The categorization into two groups allows the assumption that the socio-demographic characteristics are similar across the groups. The method involves developing a *post-hoc* quasi-experimental *post-hoc* design that compares people who are comparable in measurable characteristics, but where some of them have received treatment, while others have not.

Our treatment condition was social isolation. Using the PSM, we obtain propensity scores (PS), which measure the extent of matching of the treatment group and the control group, and also, the average effect of treatment on the treated (ATT) (Rosenbaum and Rubin, 1983; Lian et al., 2011). We adopted three PSM methods, namely, nearest-neighbor matching, radius matching, and kernel matching (Baser, 2006). The specific analysis strategies used in this study were as follows: First, we used descriptive statistical analysis to understand the mental health status of Chinese older adults, and ANOVA to explore the relationship between social isolation and mental health. Second, we used multiple linear regression models to gradually verify the correlations among subjective social isolation, aging attitude, and mental health. At the same time, the score matching method was used to explore the causal relationship between social isolation and mental health in older adults. Finally, the mediation analysis was carried out using the structural equation model, and the mediation effect of the aging attitude was verified using the bootstrap method (Hayes, 2009). The bootstrap method was used to test the intermediary effect with the random sampling replications set to 5,000, and a 95% confidence interval. All statistical analyses, as well as data cleaning, were performed in STATA software version 13.0 (StataCorp, College Station, TX, USA), and results are reported at a 95% significance threshold.

## Results

### Descriptive Statistics

Table 2 shows the descriptive statistics of the relevant variables. Approximately 13.9% of older adults suffer from family isolation, 39.9% from friend isolation, 71.2% from community isolation, and 27.3% from perceived isolation (i.e., loneliness). The average depression score among older adults was 13.5 (*SD* = 3.56), the proportion of older adults living alone was 12.2%, and those widowed were 28.6%.

**Table 2:** Descriptive statistics of the relevant variables.

**Variables**	**Percent**	**Average**	**Standard deviation (SD)**
**Independent Variables**			
Objective social isolation			
Family isolation	13.88		
Friend isolation	39.88		
Community isolation	71.20		
Subjective social isolation			
Perceived isolation	27.29		
**Mediation Variable**			
Aging attitude		19.52	5.45
**Dependent Variable**			
Depression		13.54	3.56
**Control Variables**			
Age (years)		68.88	7.35
Gender (male)	55.00		
Education			
Elementary school and below	55.60		
Junior high school	23.93		
High school and above	20.47		
Urban	64.78		
Married	28.56		
Working	20.39		
Log of income		9.21	1.58
Religious faith (Yes)	11.25		
Instrumental activity of daily living (IADL)		7.80	1.87
Cognitive ability		13.05	2.97
Number of children		2.36	0.97
Living arrangement			
Live alone	12.20		
Live only with spouse	35.72		
Live only with children	41.71		
Live with other people	10.36		

### Multiple Regression Results

To test the independent correlations of objective and subjective social isolation with depression among older adults, we established three multiple linear regression models, gradually incorporating objective social isolation, subjective social isolation, and aging attitude into the analysis model. Table 3 shows the models of the regression analysis.

**Table 3:** The linear regression results of social isolation and depression in older adults (*N* = 7,024).

**Variables**	**Depression**			
	**Model 1**	**Model 2**	**Model 3**	**VIF**
Family isolation	1.158^***^	0.798^***^	0.788^***^	1.11
	(0.116)	(0.105)	(0.100)	
Friend isolation	0.453^***^	0.449^***^	0.185^**^	1.13
	(0.083)	(0.074)	(0.071)	
Community isolation	0.038	0.079	0.050	1.02
	(0.086)	(0.077)	(0.073)	
Perceived isolation		3.365^***^	2.944^***^	1.16
		(0.082)	(0.080)	
Age (years)	−0.059^***^	−0.047^***^	−0.048^***^	1.60
	(0.007)	(0.006)	(0.006)	
Gender	0.041	−0.005	−0.036	1.16
	(0.083)	(0.075)	(0.071)	
Education				
Junior high school	−0.343^***^	−0.259^**^	−0.174^*^	1.31
	(0.103)	(0.092)	(0.088)	
High school and above	−0.468^***^	−0.347^***^	−0.235^*^	1.51
	(0.117)	(0.105)	(0.100)	
Married	0.670^***^	0.124	0.154	1.90
	(0.116)	(0.105)	(0.101)	
Working	−0.373^***^	−0.365^***^	−0.330^***^	1.24
	(0.106)	(0.095)	(0.091)	
Urban	−0.404^***^	−0.334^***^	−0.174^*^	1.48
	(0.097)	(0.087)	(0.084)	
IADL	0.429^***^	0.332^***^	0.277^***^	1.24
	(0.023)	(0.020)	(0.020)	
Religious faith	0.088	0.166	0.194	1.04
	(0.124)	(0.111)	(0.106)	
Living arrangement				
Live with spouse	−0.801^***^	−0.362^*^	−0.359^**^	3.95
	(0.159)	(0.143)	(0.136)	
Live with children	−1.114^***^	−0.510^***^	−0.449^***^	3.22
	(0.139)	(0.125)	(0.120)	
Live with other people	−0.646^***^	−0.168	−0.164	1.99
	(0.177)	(0.159)	(0.152)	
Number of children	0.156^***^	0.143^***^	0.108^**^	1.39
	(0.046)	(0.042)	(0.040)	
Cognitive ability	−0.152^***^	−0.131^***^	−0.098^***^	1.27
	(0.014)	(0.013)	(0.012)	
Log of income	−0.166^***^	−0.105^***^	−0.075^**^	1.42
	(0.029)	(0.026)	(0.025)	
Aging attitude			−0.172^***^	1.21
			(0.007)	
Constant	18.145^***^	15.961^***^	19.286^***^	
	(0.546)	(0.492)	(0.487)	
R-squared	0.186	0.345	0.403	

*VIF, variance inflation factor; IADL, instrumental activity of daily living*.

* *p < 0.05*,

** *p < 0.01*,

*** *p < 0.001*.

In model 1, depression was regressed on objective social isolation and the control variables. As can be seen from the Table, compared with older adults who did not experience family isolation, older adults experiencing family isolation had higher depression (β = 1.158, *p* < 0.001). Similarly, compared with older adults who did not experience friend isolation, older adults separated from friends had higher depression (β = 0.453, *p* < 0.001). Overall, model 1 shows that family isolation has a higher correlation with depression among older people, followed by friend isolation, while community isolation has no significant relationship.

In model 2, depression was regressed on objective isolation, subjective isolation, and the control variables. As can been seen in the second model, there was a relative attenuation in the effects for both family and friend isolation, compared with the previous model. In model 2, compared with the respective reference groups, the effect sizes for family and friend isolation reduced to (β = 0.798, *p* < 0.001) and (β = 0.449, *p* < 0.001), respectively. Furthermore, compared with older adults who did not experience perceived isolation, depression was higher (β = 3.365, *p* < 0.001) among those who experienced perceived isolation. Overall, model 2 shows that both objective and subjective social isolation have independent correlations with depression among older adults, with the latter having a higher effect size.

In model 3, depression was regressed on objective isolation, subjective isolation, aging attitude, and the control variables. It can be seen that the correlation coefficients of social isolation and depression in different dimensions were further reduced, and the explanatory power of the model increased from 34.5 to 40.3%. This result suggests that the attitude to aging may mediate the relationship between social isolation and depression. The variance inflation factor (VIF) of all models was below 4, indicating that there was no issue of multicollinearity in the models.

Among the set of control variables, education, working status, higher income, urban residency, residing with relatives or other people, and higher cognitive ability were all protective factors from depression symptoms. While on the other hand, a higher IADL score, and surprisingly, a higher number of children acted as a risk factor for depression symptoms.

### Propensity Score Matching Results

Table 4 shows the ATT results of the propensity value score matching method. It can be seen that after the matching of the sample errors of the control and treatment groups, the causal effects of family isolation, friend isolation, and perceived isolation on depression in older adults are verified. Combining the matching results of the three methods, it can be concluded that family isolation, friend isolation, and perceived isolation had ATT values of 1.3, 0.8, and 3.6, respectively, on depression.

**Table 4:** The average effect of treatment on the treated results of social isolation and mental health.

**Method of Score Matching**	**Depression**			
Propensity score matching	Family isolation	Friendisolation	Community isolation	Perceivedisolation
Nearest neighbor matching	1.240^***^	0.780^***^	0.119	3.643^***^
Radius matching	1.263^***^	0.753^***^	0.147	3.617^***^
Kernel matching	1.291^***^	0.773^***^	0.187	3.636^***^

*** *p < 0.001*.

We further analyzed the density distribution map of the control group and the treatment group before and after matching with different propensity score matching methods. The results show that the difference between the treatment group and the control group was greatly reduced after matching.

A comparison of the results of propensity score matching and multiple linear regression shows that the results of the two methods are consistent. It further illustrates the causal effect of social isolation on the mental health of older adults, specifically underscoring the fact that family isolation, friend isolation, and perceived isolation are important predictors of depression in older adults.

### Mediating Effects

We conducted a mediation analysis based on the structural equation model and used the bootstrap method to test the mediating effects of aging attitudes on the relationship between social isolation and depression in older adults.

Table 5 shows the results of the mediating effect of aging attitude. It can be seen from Table 5 that the direct effect of family isolation on depression was 0.805 (*p* = 0.000) and the indirect effect was 0.017 (*p* = 0.609); the direct effect of friend isolation on depression was 0.184 (*p* = 0.016) and the indirect effect was 0.264 (*p* = 0.000); the direct effect of perceptual isolation on depression was 2.950 (*p* = 0.000), and the indirect effect was 0.425 (*p* = 0.000). This shows that the direct effect of family isolation on depression was significant, but the indirect effect was not significant; the direct and indirect effects of friend isolation and perceived isolation on depression were significant.

**Table 5:** The mediation effect size of aging attitude in the effect of social isolation on depression.

**Path**	**Effect**	**Effect Size**	**Bootstrap SE**	***p*** **-Value**
Family isolation—Depression	Direct effect	0.805	0.1092	0.000
	Indirect effect	0.017	0.0327	0.609
Friend isolation—Depression	Direct effect	0.184	0.0762	0.016
	Indirect effect	0.264	0.0251	0.000
Community isolation—Depression	Direct effect	0.049	0.0712	0.491
	Indirect effect	0.031	0.0247	0.210
Perceived isolation—Depression	Direct effect	2.950	0.0838	0.000
	Indirect effect	0.425	0.0278	0.000

**Figure 1:**
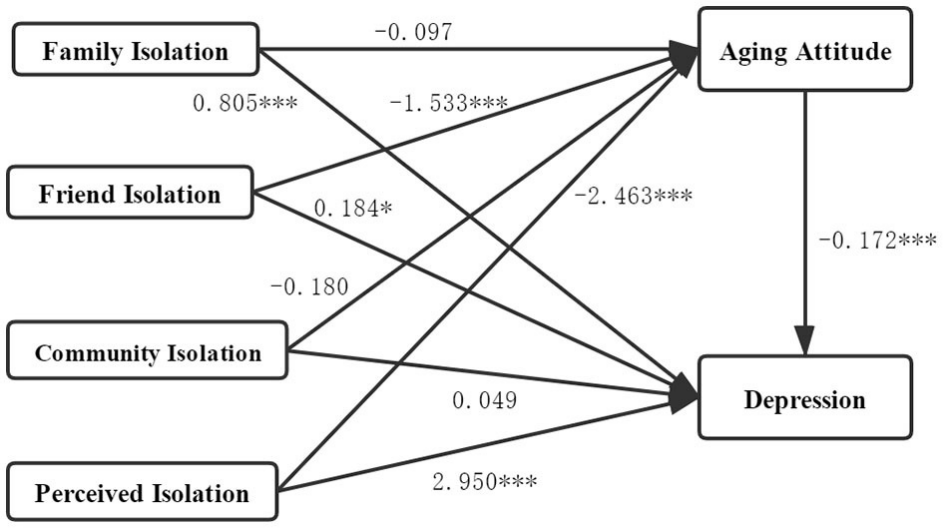
The mediation effects path of social isolation, aging attitude, and depression. ^*^p < 0.05, ^***^p < 0.001.

It can be seen that aging attitude had a significant indirect effect on the relationship between social isolation (friend isolation and perceived isolation) and depression. Figure 1 shows the path of the mediating role of aging attitude between social isolation and depression in older adults. As can be seen from Figure 1, friend isolation → aging attitude (β = −1.553, significant at *p* = 0.001), aging attitude → depression (β = −0.172, significant at p = 0.001), friend isolation → depression (β = 0.184, significant at *p* = 0.05); perceived isolation → aging attitude (β = −2.463, significant at *p* = 0.001), aging attitude → depression (β = −0.172, significant at *p* = 0.001), perceived isolation → depression (β = 2.950, significant at *p* = 0.001).

Therefore, we found two significant pathways in the relationship between social isolation, aging attitudes, and depression: Path 1, friend isolation → aging attitude → depression, where aging attitude plays an intermediary role; Path 2, perceived isolation → aging attitude → depression, where aging attitude plays an intermediary role.

The test shows that friend isolation and perceived isolation not only directly cause depression in older adults, but also indirectly cause depression among older adults through aging attitude.

## Discussion

This study examined the relationships between social isolation, attitudes toward aging, and mental health among older adults in China. The purpose was to study the effects of social isolation and aging attitudes on the mental health of older adults. Our analytical tools included multiple linear regression, structural equation modeling, and the Bootstrap technique. Against the backdrop of the research questions that guided the analysis in this study, the finding shows that (1) objective and subjective social isolation are independently and negatively associated with depression among older adults. (2) There are variations with regards to how each form of social isolation is associated with depression among older adults. Specifically, objective social isolation from family or friends had a significant effect on depression, thus underlying the importance of support from family and friends toward older people. (3) Aging attitude act as a significant mediator between social isolation and depression. To the best of our knowledge, the association between the dimensions of social isolation (in the manner we have decomposed it), and depression among Chinese older adults has never been examined. The same can also be said for the mediating role of aging attitude. The current study is therefore significant and contributes to knowledge by broadening the understanding of what dimensions of social isolation are most associated with depression among Chinese older adults. More importantly, it also provided evidence of the mediating role of aging attitude in the relationship between social isolation and depression.

Consistent with previous studies (Umberson and Montez, 2010), our findings indicate that objective and subjective social isolation have independent negative effects on the mental health of older adults. Studies have shown that when only objective social isolation variables are considered, both family and friend isolation are significantly related to depression among older adults, while community isolation was unrelated (Umberson and Montez, 2010). The introduction of the subjective social isolation variable (perceived isolation) in our study did not significantly alter this pattern, while the variable itself was also significantly and negatively associated with depression. The “net effect” for the dimensions of social isolation on depression obtained from the PSM had further underscored this finding, consistent with previous studies that have inferred that when older adults feel socially isolated, it tends to impact their mental health (Cornwell and Waite, 2009; Cacioppo et al., 2010). The measures of subjective isolation in this study may be theoretically related to that of perceived social rejection which also has a well-established relationship with depression (Slavich et al., 2010), and may also explain the strong association observed in the result.

However, it is also important to acknowledge that while our finding shows consistency with some sections of the literature, it did have dissimilarities with some others. Some research has reported that objective social isolation (family isolation and friend isolation) was unrelated to depressive symptoms and psychological distress in older adults in the United States (Taylor et al., 2018), which is inconsistent with our findings. A possible reason for the difference could be related to cultural factors. While the Chinese culture leans toward the Confucian ideology characterized by collectivism which emphasizes family togetherness and interdependencies, the Western culture which is characterized by individualism on the other hand could mean that older adults in those cultures are less reliant on relatives and friends.

Furthermore, our finding had shown in part, that isolation from family or friends is negatively associated with depression in older adults, while community isolation was not. This finding, which is also consistent with Yeung and Fung (2007), can also be similarly linked to the Chinese’s collectivist cultural context. In Chinese culture, the family is the basis of an individual’s existence as well as an important source of individual social support (Li et al., 2014). Also, friends are an important network unit for individuals to connect with society, as well as useful channels to obtain emotional support (Huxhold et al., 2014). With the adjustment to lifestyles, failing physical health, a shrunken network of friends, or social support that usually characterizes old age (Kaye and Singer, 2018), the perceived feeling of growing isolation could negatively influence the mental health of older adults. As already stated, community isolation was not associated with depression among older adults in the current study. The reason could be related to the issue of construct. Community isolation was measured from the question of whether or not the respondent engaged in some listed community activities. However, participation in such activities may depend on several factors like actual availability of the activity in their residential community, interest to participate, or physical health status. It may also be due to the composite nature in which the variable was formed. A respondent would only need to participate in one event to be considered to have experienced community participation. Whereas in reality, frequency and diversity may be necessary for a positive effect on mental health. Future studies could devise more objective tools to measure community participation.

This study found a partial mediating role of aging attitudes between social isolation and the mental health of older adults with two significant intermediary paths. First, friend isolation → aging attitude → mental health; and second, perceived isolation → aging attitude → mental health. These results show that friend isolation and perceived isolation not only have a direct impact on the mental health of older adults but also have an indirect impact on their health through aging attitudes. This, therefore, provides empirical evidence supporting the AoA model espoused in Diehl et al. (2014), which underscored the mediating role of aging attitude in the relationship between social isolation and mental health. The mechanism of such mediating role is explained by the fact that social connections could produce both emotional and instrumental support that helps older adults to form a positive attitude toward aging which may, in turn, have positive implications on their mental health. Older people will inevitably encounter decreased mental health if they encounter friend isolation in old age and have a negative attitude toward aging. Also, older adults with perceived isolation are more likely to lack interactions with society and to be more dissatisfied with the quality of their social relationships (Ryan and Willits, 2007), which increases their pessimism, and makes them have more negative attitudes toward aging, thus affecting their mental health.

Several limitations are associated with this study. First, we used cross-sectional data and as a result, it is difficult to make claims of causality. For instance, there may be an unobserved factor such as mental illness, which has a propensity for the creation of social isolation. Also, individuals may report social isolation due to a lack of bonding with relatives or friends. Second, this study focused only on depression among older adults, leaving the possibility of certain gaps in getting a more comprehensive account of the relationship between social isolation and the overall mental health of older adults. Third, the factors impacting the mental health of older adults are very complicated. For example, personality characteristics and major stressful life events such as chronic diseases or disabilities, are all important aspects that affect the mental health of older adults. The limitation of the data in capturing these variables did not allow us to control for them in our analysis. This, to some extent, may have affected the results of the current study. Forth, the questionnaire of the 2014 CLASS did not include more types of community activities. For instance, there was no question bordering on taking classes, attending senior groups or centers, or religious services. This inadequacy could lead to crude measurement of the “community isolation” variable. Also, about the data, the 2014 CLASS may be considered to have aged. However, we resorted to it because the 2016 and 2018 follow-up waves of the CLASS data are not available for public use. Besides, the AAQ was not captured in the follow-up waves according to the available reports from those surveys.

Despite these limitations, this study significantly expands the extant literature on the relationship between social isolation and the mental health of older adults in China. The use of large and nationally representative data makes our finding generalizable for a national context. Not only does the study provide evidence from the Chinese context about the mechanisms operating in the relationship between social isolation and mental health, but it also highlights the mediating role of aging attitudes.

## Conclusion

We used data from the 2014 CLASS to investigate the impact of objective isolation (family isolation, friend isolation, and community isolation), and subjective social isolation (perceived isolation) on the mental health of Chinese older adults, and the mediating effect of aging attitudes. The regression analysis indicated that objective social isolation and subjective social isolation were independently related to mental health among older adults. The mediation analysis further showed that aging attitude plays a significant mediating role between social isolation and mental health. This study has important implications for intervention from the perspective of promoting social relations and improving aging attitudes to enhance the mental health of older adults. Mental health in old age could be enhanced by reducing social isolation and a mechanism to achieve this would be to improve aging attitudes. The government, communities, relatives, and friends need to pay more attention to decreasing the social isolation of older adults, particularly in this time of COVID-19 which specifically requires social distancing and isolation as an epidemic prevention and control measure. Where physical contact is not possible, the community, relatives, and friends should strive to use modern social communication mediums to facilitate regular communication and engagement with the elderly to mitigate the negative effect of social isolation.

## Data Availability Statement

Publicly available datasets were analyzed in this study. This data can be found here: http://class.ruc.edu.cn/index.php?r=index/index&amp;hl$=$en.

## Ethics Statement

The studies involving human participants were reviewed and approved by Ethics Committee of Renmin University of China. The patients/participants provided their verbal informed consent to participate in this study.

## Author Contributions

XC: conceptualization, methodology, formal analysis, and writing—original draft. TC: methodology and formal analysis. TA: writing—review & editing. All authors contributed to the article and approved the submitted version.

## Funding

This study was supported by the Science and Technological Department of Shaanxi Province (No. 2020JM-570, PI: XC).

## Conflict of Interest

The authors declare that the research was conducted in the absence of any commercial or financial relationships that could be construed as a potential conflict of interest.

## Publisher’s Note

All claims expressed in this article are solely those of the authors and do not necessarily represent those of their affiliated organizations, or those of the publisher, the editors and the reviewers. Any product that may be evaluated in this article, or claim that may be made by its manufacturer, is not guaranteed or endorsed by the publisher.

## References

[B1] Antonucci T. C. Ajrouch K. J. Birditt K. S. (2014). The convoy model: explaining social relations from a multidisciplinary perspective. Gerontologist 54, 82–92. 10.1093/geront/gnt118 24142914 PMC3894851

[B2] Antonucci T. C. Akiyama H. (1995). Convoys of Social Relations: Family and Friendships Within a Life Span Context. Westport: Greenwood Press/Greenwood Publishing Group.

[B3] Baser O. (2006). Too much ado about propensity score models? Comparing methods of propensity score matching. Value Health 9, 377–385. 10.1111/j.1524-4733.2006.00130.x 17076868

[B4] Berkman L. F. Glass T. Brissette I. Seeman T. E. (2000). From social integration to health: Durkheim in the new millennium. Soc. Sci. Med. 51, 843–857. 10.1016/S0277-9536(00)00065-4 10972429

[B5] Cacioppo J. T. Cacioppo S. (2014). Social relationships and health: the toxic effects of perceived social isolation. Soc. Pers. Psychol. Compass 8, 58–72. 10.1111/spc3.12087 24839458 PMC4021390

[B6] Cacioppo J. T. Hawkley L. C. Thisted R. A. (2010). Perceived social isolation makes me sad: 5-year cross-lagged analyses of loneliness and depressive symptomatology in the Chicago health, aging, and social relations study. Psychol. Aging 25, 453–463. 10.1037/a0017216 20545429 PMC2922929

[B7] Chang Q. Sha F. Chan C. H. Yip P. Harris K. M. (2018). Validation of an abbreviated version of the Lubben Social Network Scale (“LSNS-6”) and its associations with suicidality among older adults in China. PLoS ONE 13:e0201612. 10.1371/journal.pone.0201612 30071067 PMC6072030

[B8] Chiu H. Lee H. C. Chung W. S. Kwong P. K. (1994). Reliability and validity of the cantonese version of Mini-Mental State Examination-A preliminary study. Hong Kong J. Psychiatry 4, 25–28.

[B9] Cho J. H. Olmstead R. Choi H. Carrillo C. Seeman T. E. Irwin M. R. (2019). Associations of objective versus subjective social isolation with sleep disturbance, depression, and fatigue in community-dwelling older adults. Aging Ment. Health 23, 1130–1138. 10.1080/13607863.2018.1481928 30284454 PMC6447478

[B10] Cohen S. (2004). Social relationships and health. Am. Psychol. 59, 676–684. 10.1037/0003-066X.59.8.676 15554821

[B11] Cornwell E. Y. Waite L. J. (2009). Social disconnectedness, perceived isolation, and health among older adults. J. Health Soc. Behav. 50, 31–48. 10.1177/002214650905000103 19413133 PMC2756979

[B12] Courtin E. Knapp M. (2017). Social isolation, loneliness and health in old age: a scoping review. Health Soc. Care Commun. 25, 799–812. 10.1111/hsc.12311 26712585

[B13] Coyle C. E. Dugan E. (2012). Social isolation, loneliness and health among older adults. J. Aging Health 24, 1346–1363. 10.1177/0898264312460275 23006425

[B14] Diehl M. Wahl H. W. Barrett A. E. Brothers A. F. Miche M. Montepare J. M.. (2014). Awareness of aging: theoretical considerations on an emerging concept. Dev. Rev. 34, 93–113. 10.1016/j.dr.2014.01.001 24958998 PMC4064469

[B15] Folstein M. F. Folstein S. E. McHugh P. R. (1975). “Mini-mental state”: a practical method for grading the cognitive state of patients for the clinician. J. Psychiatr. Res. 12,189–198. 10.1016/0022-3956(75)90026-6 1202204

[B16] Ge L. Yap C. W. Ong R. Heng B. H. (2017). Social isolation, loneliness and their relationships with depressive symptoms: a population-based study. PLoS ONE 12:e0182145. 10.1371/journal.pone.0182145 28832594 PMC5568112

[B17] Gerino E. Rollè L. Sechi C. Brustia P. (2017). Loneliness, resilience, mental health, and quality of life in old age: a structural equation model. Front. Psychol. 8:2003. 10.3389/fpsyg.2017.02003 29184526 PMC5694593

[B18] Hayes A. F. (2009). Beyond Baron and Kenny: statistical mediation analysis in the new millennium. Commun. Monogr. 76, 408–420. 10.1080/03637750903310360

[B19] Holt-Lunstad J. Smith T. B. Baker M. Harris T. Stephenson D. (2015). Loneliness and social isolation as risk factors for mortality: a meta-analytic review. Perspect. Psychol. Sci. 10, 227–237. 10.1177/1745691614568352 25910392

[B20] Holt-Lunstad J. Smith T. B. Layton J. B. (2010). Social relationships and mortality risk: a meta-analytic review. PLoS Med. 7:e1000316. 10.1371/journal.pmed.1000316 20668659 PMC2910600

[B21] Huang G. Duan Y. Guo F. Chen G. (2020). Prevalence and related influencing factors of depression symptoms among empty-nest older adults in China. Arch. Gerontol. Geriatr. 91:104183. 10.1016/j.archger.2020.104183 32721660

[B22] Hughes M. E. Waite L. J. Hawkley L. C. Cacioppo J. T. (2004). A short scale for measuring loneliness in large surveys - Results from two population-based studies. Res. Aging 26, 655–672. 10.1177/0164027504268574 18504506 PMC2394670

[B23] Huxhold O. Miche M. Schuz B. (2014). Benefits of having friends in older ages: differential effects of informal social activities on well-being in middle-aged and older adults. J. Gerontol. Ser. B. Psychol. Sci. Soc. Sci. 69, 366–375. 10.1093/geronb/gbt029 23682165

[B24] Jang Y. Poon L. W. Kim S.-Y. Shin B.-K. (2004). Self-perception of aging and health among older adults in Korea. J. Aging Stud. 18, 485–496. 10.1016/j.jaging.2004.06.001

[B25] Kaye L. W. (2017). Older adults, rural living, and the escalating risk of social isolation. Public Policy Aging Rep. 27, 139–144. 10.1093/ppar/prx029

[B26] Kaye L. W. Singer C. (2018). Social Isolation of Older Adults: Strategies to Bolster Health and Well-Being. New York, NY: Springer Publishing Company. 10.1891/9780826146991

[B27] Korkmaz Aslan G. Kulakci Altinta S. H. Ozen Cinar I. Veren F. (2019). Attitudes to ageing and their relationship with quality of life in older adults in Turkey. Psychogeriatrics 19, 157–164. 10.1111/psyg.12378 30338609

[B28] Laidlaw K. (2010). Attitudes to ageing and expectations for filial piety across Chinese and British cultures: a pilot exploratory evaluation. Aging Ment. Health 14, 283–292. 10.1080/13607860903483060 20425647

[B29] Laidlaw K. Power M. J. Schmidt S. Group W.-O. (2007). The Attitudes to Ageing Questionnaire (AAQ): development and psychometric properties. Int. J. Geriatr. Psychiatry 22, 367–379. 10.1002/gps.1683 17051535

[B30] Lamont R. A. Nelis S. M. Quinn C. Clare L. (2017). Social support and attitudes to aging in later life. Int. J. Aging Hum. Dev. 84, 109–125. 10.1177/0091415016668351 27655953

[B31] Laugesen K. Baggesen L. M. Schmidt S. A. J. Glymour M. M. Lasgaard M. Milstein A.. (2018). Social isolation and all-cause mortality: a population-based cohort study in Denmark. Sci. Rep. 8:4731. 10.1038/s41598-018-22963-w 29549355 PMC5856842

[B32] Lawton M. P. Brody E. M. (1988). Instrumental Activities of Daily Living (IADL) scale – self -rated version. Psychopharmacol. Bull. 24, 789–791. 3249786

[B33] Li H. Ji Y. Chen T. (2014). The roles of different sources of social support on emotional well-being among Chinese elderly. PLoS ONE 9, 1–8. 10.1371/journal.pone.0090051 24594546 PMC3940715

[B34] Lian Y. Su Z. Gu Y. (2011). Evaluating the effects of equity incentives using PSM: evidence from China. Front. Bus. Res. China 5, 266–290. 10.1007/s11782-011-0131-6

[B35] Lim L. L. Kua E. H. (2011). Living alone, loneliness, and psychological well-being of older persons in singapore. Curr. Gerontol. Geriatr. Res. 2011:673181. 10.1155/2011/673181 21969827 PMC3182578

[B36] Liu D. Xi J. Hall B. J. Fu M. Zhang B. Guo J.. (2020). Attitudes toward aging, social support and depression among older adults: difference by urban and rural areas in China. J. Affect. Disord. 274, 85–92. 10.1016/j.jad.2020.05.052 32469837

[B37] Lubben J. Blozik E. Gillmann G. Iliffe S. Kruse W. v. R.. (2006). Performance of an abbreviated version of the Lubben Social Network Scale among three european community-dwelling older adult populations. Gerontologist 46, 503–513. 10.1093/geront/46.4.503 16921004

[B38] Menec V. H. Newall N. E. Mackenzie C. S. Shooshtari S. Nowicki S. (2019). Examining individual and geographic factors associated with social isolation and loneliness using Canadian Longitudinal Study on Aging (CLSA) data. PLoS ONE 14:e0211143. 10.1371/journal.pone.0211143 30707719 PMC6358157

[B39] National Survey Research Center (2014). 2014 Chinese Longitudinal Aging Social Survey (CLASS) Final Report. Beijing: National Survey Research Center, Renmin University of China.

[B40] Qin X. Wang S. Hsieh C. R. (2016). The prevalence of depression and depressive symptoms among adults in China: Estimation based on a national household survey. China Econ. Rev 51, 271–282. 10.1016/j.chieco.2016.04.001

[B41] Robins L. M. Hill K. D. Finch C. F. Clemson L. Haines T. (2018). The association between physical activity and social isolation in community-dwelling older adults. Aging Ment. Health 22, 175–182. 10.1080/13607863.2016.1242116 27736192

[B42] Rosenbaum P. R. Rubin D. B. (1983). The central role of the propensity score in observational studies for causal effects. Biometrika 70, 41–55. 10.1093/biomet/70.1.41

[B43] Ryan A. K. Willits F. K. (2007). Family ties, physical health, and psychological well-being. J. Aging Health 19, 907–920. 10.1177/0898264307308340 18165288

[B44] Santini Z. I. Koyanagi A. Tyrovolas S. Mason C. Haro J. M. (2015). The association between social relationships and depression: a systematic review. J. Affect. Disord. 175, 53–65. 10.1016/j.jad.2014.12.049 25594512

[B45] Santini Z. I. Koyanagi A. I. Tyrovolas S. Haro J. M. Koushede V. (2017). The association of social support networks and loneliness with negative perceptions of ageing: evidence from the Irish Longitudinal Study on Ageing (TILDA). Ageing Soc. 39, 1070–1090. 10.1017/S0144686X17001465

[B46] Schrempft S. Jackowska M. Hamer M. Steptoe A. (2019). Associations between social isolation, loneliness, and objective physical activity in older men and women. BMC Public Health 19:74. 10.1186/s12889-019-6424-y 30651092 PMC6335852

[B47] Shaw J. G. Farid M. Noel-Miller C. Joseph N. Houser A. Asch S. M.. (2017). Social isolation and medicare spending: among older adults, objective social isolation increases expenditures while loneliness does not. J. Aging Health 29, 1119–1143. 10.1177/0898264317703559 29545676 PMC5847278

[B48] Silverstein M. Cong Z. Li S. (2006). Intergenerational transfers and living arrangements of older people in rural China: consequences for psychological well-Being. J. Gerontol. Ser. B. Psychol. Sci. Soc. Sci. 61, S256–S266. 10.1093/geronb/61.5.S256 16960239

[B49] Slavich G. M. O’Donovan A. Epel E. S. Kemeny M. E. (2010). Black sheep get the blues: a psychobiological model of social rejection and depression. Neurosci. Biobehav. Rev. 35, 39–45. 10.1016/j.neubiorev.2010.01.003 20083138 PMC2926175

[B50] Taylor H. O. Taylor R. J. Nguyen A. W. Chatters L. (2018). Social isolation, depression, and psychological distress among older adults. J. Aging Health 30, 229–246. 10.1177/0898264316673511 28553785 PMC5449253

[B51] Thompson M. G. Heller K. (1990). Facets of support related to well-being: quantitative social isolation and perceived family support in a sample of elderly women. Psychol. Aging 5, 535–544. 10.1037/0882-7974.5.4.535 2278677

[B52] Tong A. Y. C. Man D. W. K. (2002). The validation of the Hong Kong Chinese version of the Lawton instrumental activities of daily living scale for institutionalized elderly persons. OTJR 22, 132–142. 10.1177/153944920202200402

[B53] Tong H. Lai D. W. L. (2016). Social exclusion and health among older Chinese in Shanghai, China. Asia Pac. J. Soc. Work Dev. 26, 120–141. 10.1080/02185385.2016.1219272

[B54] Umberson D. Crosnoe R. Reczek C. (2010). Social relationships and health behavior across life course. Annu. Rev. Sociol. 36, 139–157. 10.1146/annurev-soc-070308-120011 21921974 PMC3171805

[B55] Umberson D. Montez J. K. (2010). Social relationships and health: a flashpoint for health policy. J. Health Soc. Behav. 51, S54–S66. 10.1177/0022146510383501 20943583 PMC3150158

[B56] United Nations and DESA. (2019). Population Division (2019). New York, NY: World Population Prospects 2019.

[B57] Valtorta N. Hanratty B. (2012). Loneliness, isolation and the health of older adults: do we need a new research agenda? J. R. Soc. Med. 105, 518–522. 10.1258/jrsm.2012.120128 23288086 PMC3536512

[B58] Valtorta N. K. Kanaan M. Gilbody S. Hanratty B. (2016). Loneliness, social isolation and social relationships: what are we measuring? A novel framework for classifying and comparing tools. BMJ Open 6:e010799. 10.1136/bmjopen-2015-010799 27091822 PMC4838704

[B59] Wang D. Laidlaw K. Power M. J. Shen J. (2009). Older people’s belief of filial piety in China: Expectation and non-expectation. Clin. Gerontol. 33, 21–38. 10.1080/07317110903347771

[B60] Wang J. Lloyd-Evans B. Giacco D. Forsyth R. Nebo C. Mann F.. (2017). Social isolation in mental health: a conceptual and methodological review. Soc. Psychiatry Psychiatr. Epidemiol. 52, 1451–1461. 10.1007/s00127-017-1446-1 29080941 PMC5702385

[B61] Wu F. Sheng Y. (2020). Differences in social isolation between young and old elderly in urban areas of Beijing, China: a cross-sectional study. Int. J. Nurs. Sci. 7, 49–53. 10.1016/j.ijnss.2019.11.003 32099859 PMC7031121

[B62] Wurm S. Diehl M. Kornadt A. E. Westerhof G. J. Wahl H.-W. (2017). How do views on aging affect health outcomes in adulthood and late life? Explanations for an established connection. Dev. Rev. 46, 27–43. 10.1016/j.dr.2017.08.002 33927468 PMC8081396

[B63] Yeung G. T. Fung H. H. (2007). Social support and life satisfaction among Hong Kong Chinese older adults: family first? Eur. J. Ageing 4, 219–227. 10.1007/s10433-007-0065-1 28794791 PMC5546368

[B64] Yu B. Steptoe A. Chen Y. Jia X. (2020). Social isolation, rather than loneliness, is associated with cognitive decline in older adults: The China Health and Retirement Longitudinal Study. Psychol. Med. 1–8. 10.1017/S0033291720001014. [Epub ahead of print]. 33478615

[B65] Zebhauser A. Hofmann-Xu L. Baumert J. Hafner S. Lacruz M. E. Emeny R. T.. (2014). How much does it hurt to be lonely? Mental and physical differences between older men and women in the KORA-Age Study. Int. J. Geriatr. Psychiatry 29, 245–252. 10.1002/gps.3998 23804458

